# Time-lapse mesoscopy of *Candida albicans* and *Staphylococcus aureus* dual-species biofilms reveals a structural role for the hyphae of *C. albicans* in biofilm formation

**DOI:** 10.1099/mic.0.001426

**Published:** 2024-01-23

**Authors:** Katherine J. Baxter, Fiona A. Sargison, J. Ross Fitzgerald, Gail McConnell, Paul A. Hoskisson

**Affiliations:** ^1^​ Strathclyde Institute of Pharmacy and Biomedical Sciences, University of Strathclyde, 161 Cathedral Street, Glasgow, G4 0RE, UK; ^2^​ The Roslin Institute, University of Edinburgh, Easter Bush Campus, Edinburgh, EH25 9RG, UK

**Keywords:** *Candida albicans*, *Staphylococcus aureus*, biofilm, co-infection

## Abstract

Polymicrobial infection with *Candida albicans* and *Staphylococcus aureus* may result in a concomitant increase in virulence and resistance to antimicrobial drugs. This enhanced pathogenicity phenotype is mediated by numerous factors, including metabolic processes and direct interaction of *S. aureus* with *C. albicans* hyphae. The overall structure of biofilms is known to contribute to their recalcitrance to treatment, although the dynamics of direct interaction between species and how it contributes to pathogenicity is poorly understood. To address this, a novel time-lapse mesoscopic optical imaging method was developed to enable the formation of *C. albicans*/*S. aureus* whole dual-species biofilms to be followed. It was found that yeast-form or hyphal-form *C. albicans* in the biofilm founder population profoundly affects the structure of the biofilm as it matures. Different sub-populations of *C. albicans* and *S. aureus* arise within each biofilm as a result of the different *C. albicans* morphotypes, resulting in distinct sub-regions. These data reveal that *C. albicans* cell morphology is pivotal in the development of global biofilm architecture and the emergence of colony macrostructures and may temporally influence synergy in infection.

## Introduction

Biofilms are communities of micro-organisms within a self-generated extracellular matrix [1, 2]. Organisms within biofilms exhibit enhancement of survival against deleterious agents due to the complex matrix of secreted proteins, lipids, extracellular DNA and polysaccharides that surround them [3]. This complex interaction of organisms and matrix components provides protection against biotic and abiotic stress that is not available to planktonic cells [4, 5]. In healthcare situations, biofilm growth can promote survival through limiting diffusion of, sequestering and inactivating antimicrobial agents, along with resistance to mechanical removal [6–8]. Biofilms may also promote the evolution of antimicrobial resistance through reducing effective concentrations of antimicrobial agents [9, 10] and by facilitating horizontal gene transfer in cells in close contact [11].

The dimorphic fungus *Candida albicans* and the bacterium *Staphylococcus aureus* are two prominent opportunistic pathogenic members of the human skin microflora [12–14]. Severity of disease caused by *C. albicans* or *S. aureus* can vary from mild cutaneous infection [15] to systemic infection with multiple tissue involvement [16]. *C. albicans* and *S. aureus* often coinfect [17, 18], and become associated with indwelling medical device infections and failure of orthopaedic implants [19–22]. Several studies have highlighted the propensity of *C. albicans* to enhance *S. aureus* virulence due to activation of toxin production [23, 24], increased biofilm growth through prostaglandin production [25] and enhanced antimicrobial resistance due to *C. albicans* augmenting the biofilm matrix [26]. *C. albicans* may also facilitate dissemination of *S. aureus* through adherence to hyphae, driving systemic infection [27].

Remarkably, biofilm formation by *S. aureus* alone is relatively poor [28]. Yet, in mixed-species biofilms cell–cell interactions have been shown to enable biofilm formation, with *C. albicans* adhesins playing a role in local neighbourhood interactions, where *C. albicans* acts as a scaffold for deposition of *S. aureus* cells [29]. The formation of microcolonies of *S. aureus,* coated in secreted matrix derived from *C. albicans*, has been proposed within these mixed-species biofilms as the mechanism for increasing antimicrobial resistance in *S. aureus* but not *C. albicans* [29]. Given the role played by *C. albicans* in nucleating *S. aureus* in mixed biofilms and the association of the dimorphic switch with virulence and host interaction response [30], the role played by *C. albicans* cell morphotypes in biofilm structure and dynamics may be vital to understanding mixed biofilm dynamics, yet is currently poorly understood.

To overcome the resolution limitations and inability to resolve macrostructures associated with traditional optical microscopy, the Mesolens can be exploited to image whole mixed-species biofilms. The Mesolens bridges the gap in scale between a macrophotography setup and a light microscope [31], with an objective providing the unusual combination of low magnification (4×) and high numerical aperture (0.47). Imaging of specimens up to 6×6×3 mm in size can be achieved with a lateral resolution of 700 nm and an axial resolution of 7 µm. Multi-channel imaging is also possible using spectral filtering. Applications of the Mesolens in microbiology have previously revealed the presence of intracolony channels in *Escherichia coli* and their adaptive response to nutrient availability [32, 33], but this work has been limited to single-species biofilms only.

To determine the role played by *Candida* morphotypes in the structure and dynamics of mixed-species biofilms, dual-species biofilms containing either yeast-form or hyphal-form *C. albicans* with *S. aureus* were studied using the first instance of time-lapse mesoscopy. This work demonstrates the emergence of distinct subpopulations within of each of the species in the mixed-species biofilms and that the nature of these is governed by the morphotype of the *Candida* present, leading to unique and remarkable dynamics and features of the mixed *C. albicans/S. aureus* biofilms.

## Methods

### Generation of a constitutively expressing mCherry *S. aureus* isolate

Chromosomal integration of mCherry into *S. aureus* strain N315 [34] was performed using the methodology outlined by De Jong *et al*. [35]. Briefly, the mCherry harbouring plasmid pRN111 was isolated from *E. coli* strain DC10B and electroporated into electrocompetent *S. aureus* N315. Transformants containing the plasmid were selected on tryptone soy agar (TSA) containing 10 µg ml^−1^ chloramphenicol (Cam, Sigma) and incubated for 24–48 h at 30 °C. Single crossover events through homologous recombination were generated via two rounds of plating onto TSA containing 7.5 µg ml^−1^ Cam followed by incubation at 42 °C overnight. Single recombinants were inoculated into 5 ml tryptone soy broth without antibiotics and incubated at 30 °C, 200 r.p.m. overnight. Cultures were diluted 1 in 1000 for five passages to promote double crossover events. Planktonic bacteria were plated onto TSA with 200 ng ml^−1^ anhydrotetracycline (ATc, Sigma) and incubated overnight at 37 °C. Integrated mutants were screened by patch plating colonies onto both TSA with and without 10 µg ml^−1^ Cam. Colonies that demonstrated the correct phenotype were screened for successful mCherry integration by PCR using primers mCherry_OUT_F: TACGACAATTCAAGAGCTTGC and mCherry_OUT_R: GAGTAAGCCAGAACAGTTCC alongside whole-genome sequencing (MicrobesNG, Birmingham UK).

### Strains and growth conditions

Fluorescent derivatives of *C. albicans* [pACT1-GFP [36]; constitutive green fluorescent protein (GFP) expressing] and *S. aureus* (N315 constitutive mCherry expressing, described above) were cultured on yeast extract peptone dextrose (YPD) and lysogeny broth (LB) agar, respectively.

Seed cultures for dual-species biofilm were generated from single colonies of each species inoculated as monocultures in 5 ml of lysogeny broth (Merck Life Science, UK) supplemented with 0.2 % glucose. Those 5 ml overnight cultures destined for hyphal-form biofilms were incubated for 16 h with shaking at 37 °C /250 r.p.m., resulting in hyphal formation within the *C. albicans* stationary phase culture.

Overnight cultures destined for yeast-form biofilms underwent a dilution step in culture set-up to allow *C. albicans* to reach stationary phase prior to hyphal formation. Seed cultures for yeast-form biofilms were initially inoculated identically to those for hyphal-form biofilms, but, directly after inoculation, those 5 ml monocultures were vortexed to disperse cells, and immediately used to inoculate a second set of 5 ml lysogeny broth at a dilution of 500-fold for *C. albicans* monocultures, and 1000-fold for *S. aureus* monocultures. These diluted monocultures were used as yeast-form seed cultures, and were incubated for 16 h with shaking at 37 °C/250 r.p.m. After the 16 h, viable cells (colony-forming units; c.f.u.) were determined from each culture by plating prior to the culture mixing step (described below). Vigorous agitation of seed cultures was carried out prior to c.f.u. serial dilution steps to prevent distortion of viable cell counts by hyphal aggregation.

### Time-lapse mesoscopy of biofilm growth

Biofilm growth conditions were adapted from the colony spreading biofilm methodology described by Kaito and Sekimizu [37]. The 16 h cultures (see above) were mixed and 2 µl was spotted onto LB agar mesoscopy mounts (see below), air-dried for 10 min and immediately imaged to produce time zero images of initial cell deposition. Initial spots consist of averages of 3.83×10^9^ c.f.u. ml^−1^ *S*. *aureus* and 1.3×10^8^ c.f.u. ml^−1^ *C*. *albicans*. After initial *t*=0 imaging, samples were incubated at 37 °C for 12 h with removal at 3, 6, 9 and 12 h post-inoculation for time-lapse imaging of biofilm formation. After imaging at the indicated time point, samples were returned to the incubator.

### Development of a specimen mount for time-lapse mesoscopy

**Fig. 1.**
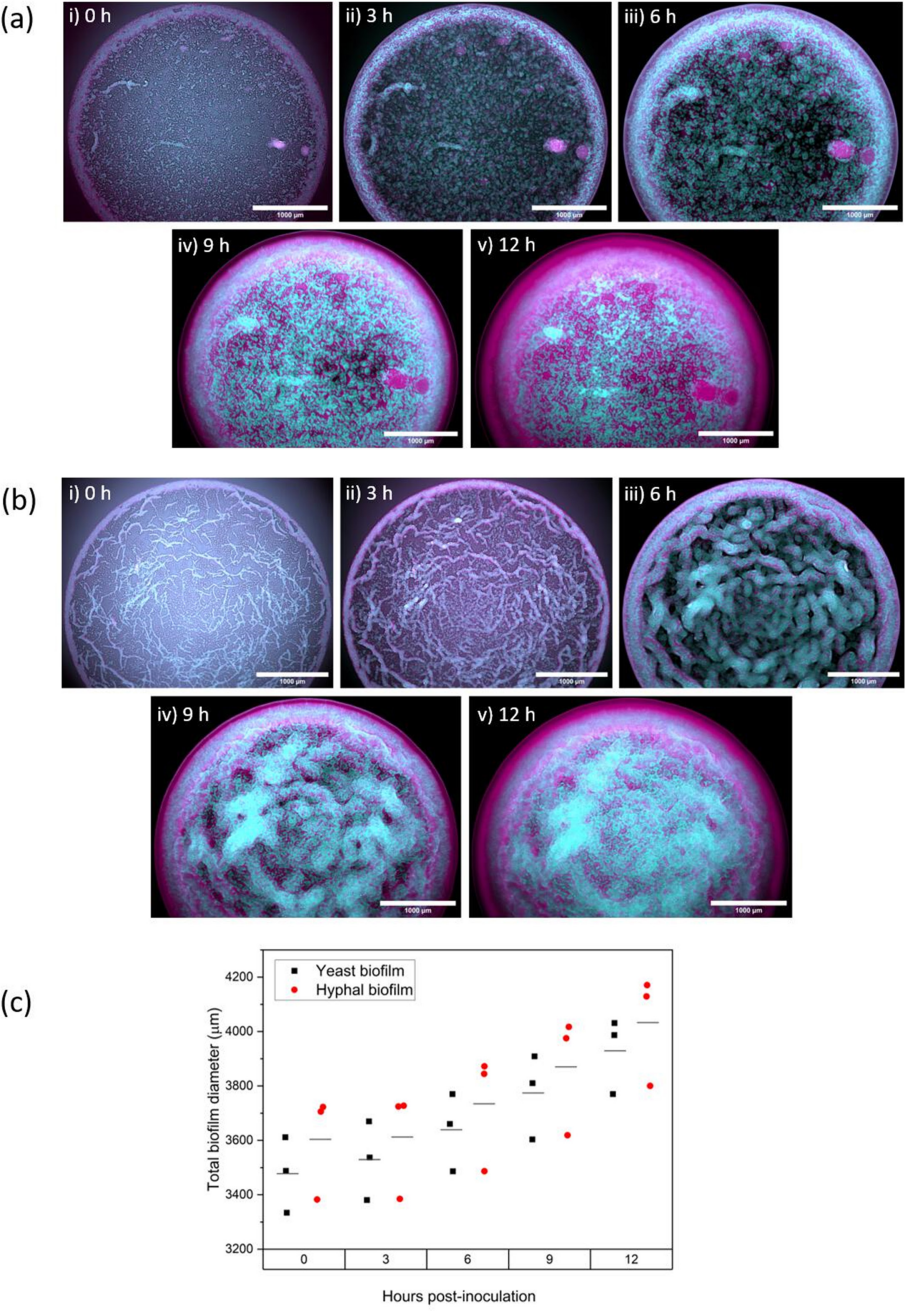
Time-lapse optical mesoscopic imaging. 12 h time-lapse mesoscopy of *C. albicans* pACT-1 GFP (cyan)*/S. aureus* N315 mCherry (magenta) dual-species biofilms. (a) Yeast-form *C. albicans/S. aureus*. (b) Hyphal-form *C. albicans/S. aureus*. (**i**) Initial deposition at *t*=0; (ii) t3, 3 h post-inoculation; (iii) t6, 6 h post-inoculation; (iv) t9, 9 h post-inoculation; (**v**) t12, 12 h post-inoculation. Images are maximum-intensity z-projections. Scale bar, 1000 µm. (c) Average diameter of both yeast-form and hyphal-form biofilms at each time point.

Imaging of samples for optical mesocopy is best achieved in optically matched liquid [31]; however, colonies of both *C. albicans* and *S. aureus* dissipate when immersed in liquid, negating the use of previously established Mesolens imaging methods [32]. Imaging through a thin layer of LB agar solidified on a glass coverslip permitted biofilms to be viewed through the base of the sample (Fig. 1a, b), facilitating water immersion for best image quality, and allowing biofilm formation to be followed over time. (Fig. S1, available in the online version of this article – image of mount). Samples were prepared and followed over time as described above.

### Sugru dam manifold manufacture and preparation

Sugru mouldable glue (Sugru, Amazon) was moulded into a long cylinder and adhered to a glass coverslip 70×70 mm type 1.5 0107999098 (Marienfeld, Lauda-Koenigshofen, Germany) to form a circular dam 45 mm in diameter and approximately 10 mm in height. After adhesion to the glass coverslip, the circular Sugru dam was left to cure for 24 h prior to use as per the manufacturer’s instructions. Manifolds were sterilized by immersion in 70 % (v/v) ethanol and then air-dried and exposed to UV light for 15 min. The interior of the dam was filled with molten LB agar supplemented with 0.2 % glucose to a depth of 2 mm, and left to solidify. Manifolds were prepared on the day of imaging, prior to culture mixing.

### Time-lapse mesoscopy

Widefield epi-fluorescent mesoscopic imaging was performed by modification of the method described by Bottura *et al.* [33]. Excitation of fluorescent proteins was achieved with a CoolLED pE-4000 LED (CoolLED, UK) at excitation/emission wavelengths of 490/525±20 nm for GFP and 585/635±20 nm for mCherry. High-resolution images were captured using a chip-shifting camera sensor (VNP-29MC; Vieworks, Anyang, Republic of Korea), which recorded images by shifting a 29 megapixel charge-coupled device (CCD) chip in a 3×3 array [38]. In this mode, the sampling rate was 4.46 pixels µm^−1^: this corresponded to a 224 nm pixel size, satisfying Nyquist sampling criteria.

Mesoscopy was performed with the correction collars of the Mesolens set for water immersion to match the refractive index of LB agar (1.33 vs 1.34, respectively). To capture the three-dimensional spatial distribution of *C. albicans* and *S. aureus* as biofilms developed over time, z-stacks of biofilms were acquired over a 12 h time period. To obtain z-stacks, the specimen was moved in the axial direction in 5 µm increments using a computer-controlled specimen stage (Optiscan III, Prior Scientific). Biofilms were incubated at 37 °C with imaging performed at time 0 h and at 3 h intervals thereafter.

### Image analysis

Three independent replicates of hyphal-form and yeast-form biofilms were imaged as described above and used in analyses. Z-stacks were converted to maximum-intensity projections using the FIJI image processing software (ImageJ, version 2.1.0/I.53c) [39]. Measurements of *Candida* hyphal width, biofilm core diameter, halo width and total diameter were taken with the measurement feature of FIJI. Three separate measurements of core diameter, halo width and total diameter of each biofilm were taken to provide a representative value for each macrostructure. To understand the degree of association of *S. aureus* with *C. albicans* in biofilm structures, colocalization of the strains was undertaken. Selection of the core region of the biofilm from the halo regions was performed by merging GFP and mCherry channels into a two-colour image, cropping the total available core area within the halo and splitting the cropped image back into separate channels. Each resulting colour channel was subjected to image thresholding, converted to a binary image and subsequently analysed with the Just another Colocalization Plugin (JACoP) [40] to determine colocalization of *C. albicans* and *S. aureus* in biofilms. Pearsons’ correlation coefficient (PCC) and Manders’ overlap coefficients (MOC) were used to statistically assess the degree of colocalization. PCC analysis of core regions used the intensities of each signal (mCherry or GFP) for a given pixel across the appropriate colour channel to calculate the correlation of signal intensity of each channel with the other. A PCC value of zero indicates there is no association between the two signals. Increasing positive values indicate increasing levels of colocalization. MOC is a measure of the fraction of overlapping signals, the M1 coefficient is the degree to which the GFP signal overlaps with mCherry signal and the M2 coefficient is the degree to which the mCherry signal overlaps with GFP. Dual-channel images were generated using QiTissue software (version 1.2.1 pre-release version, Quantitative Imaging Systems LLC). To identify whether hyphae were components of the *C. albicans* projections into the *S. aureus* peripheral halo, plot profiles of projections were derived from the FIJI line measurement tool and the resulting grey value curves were analysed using the full width at half maximum (FWHM) principle, where the width of the curve is measured between the *y*-axis points that are half the height of the curve. Values were calculated using the FWHM.ijm macro available on GitHub. (https://gist.github.com/lacan/45f865b5a38d7c3a96a7cd8b25923407). Assessment of *C. albicans* and *S. aureus* growth in both biofilms was indirectly quantified by measurement of percentage changes in fluorescence intensity. Corrected total fluorescence of 525 nm (*C. albicans*) and 635 nm (*S. aureus*) images at 3 and 12 h were calculated by subtracting the product of biofilm area and mean background fluorescence from biofilm integrated density, and the resultant percentage increase in fluorescence intensity was derived to compare biomass increase. The 3 h time point was chosen as the initial reference point to allow cells to recover from stationary phase after overnight culture.

### Antibiotic susceptibility testing of biofilms

Decreasing concentrations of vancomycin (Fisher Scientific, UK) were generated by the broth dilution method [41] to establish the lowest concentration of vancomycin required to prevent growth of planktonic *S. aureus* in overnight cultures (3.125 µg ml^−1^). Once identified, a variation of the protocol undertaken by Adam *et al.* [42] was used to test the susceptibility of planktonic seed cultures of *S. aureus* grown in the presence of yeast-form and hyphal-form *Candida* cells after 5 h exposure to vancomycin. Planktonic cultures (16 h) were treated with vancomycin (3.125 µg ml^−1^) and incubated at 37 °C with shaking for 5 h. In the case of dual-species biofilms, 6 h biofilms were treated with 4 µl of LB containing 3.125 µg ml^−1^ placed onto the top of each biofilm and allowed to dry before being incubated at 37 °C for 5 h. After exposure, biofilms were removed from agar by scalpel and placed into an Eppendorf tube, resuspended v/v into 1 ml of LB by mixing. The resulting cell suspension was used to determine viability by c.f.u. count on LB agar containing fluconazole to prevent *C.albicans* growth. All sampling was performed in triplicate.

## Results

### Common features of biofilms of both *Candida* morphotypes are the result of fluid dynamics

Initial seeding of the biofilm cultures resulted in founder populations of *C. albicans* and *S*. aureus in yeast-form biofilms averaging 3.65×10^9^ c.f.u. ml^−1^ *S*. *aureus* and 1.34×10^8^ c.f.u. ml^−1^ *C*. *albicans,* and 4×10^9^ c.f.u. ml^−1^ *S*. *aureus* and 1.28×10^8^ c.f.u. ml^−1^ *C*. *albicans* in hyphal-form biofilms. Evaporation of the mixed cell inoculation mixture resulted in cells being deposited in the archetypal ‘coffee ring’ structure due to fluid dynamics associated with evaporation of liquid drops [43, 44]. This causes the formation of a ring of *C. albicans* and *S. aureus* cells (halo), surrounding a ‘core’ of sparsely deposited cells. This was found in cultures established with both yeast-form and hyphal-form *Candida* in the cell mixture (Fig. 1a, b).

### Macrostructures emerge in mixed-species biofilms containing either *Candida* morphotype

**Fig. 2.**
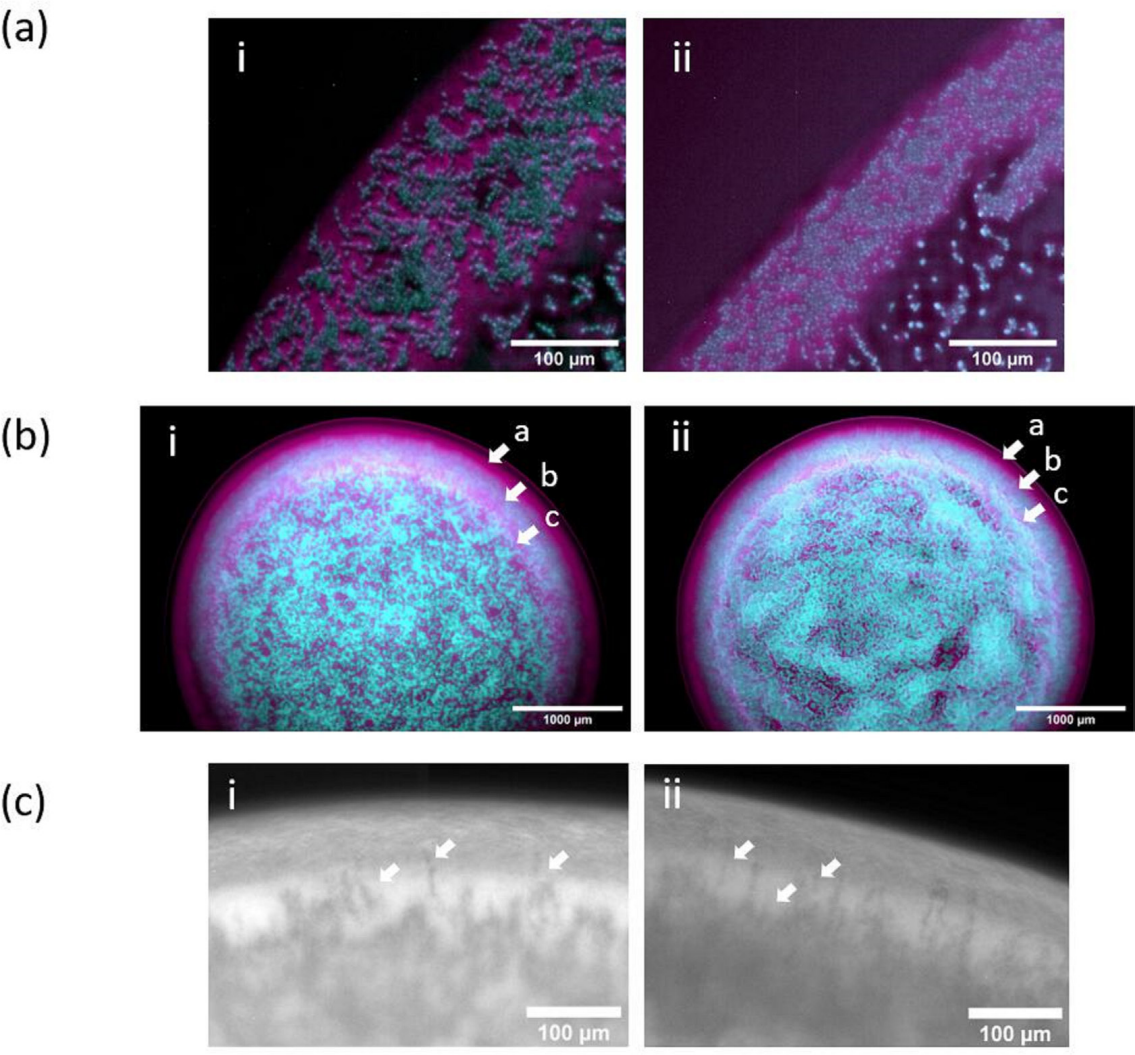
Yeast-form and hyphal-form halo structures of *C. albicans* pACT-1 GFP (cyan)*/S. aureus* N315 mCherry (magenta) dual-species biofilms. (a) Digital zoom of halo illustrating similar cellular form and composition at initial deposition (*t*=0 h) in (**i**) yeast-form *C. albicans/S. aureus* biofilm and (ii) hyphal-form biofilm. (b) Comparison of species banding patterns in halos of (**i**) yeast-form *C. albicans/S. aureus* biofilm and (ii) hyphal-form biofilm 12 h post-inoculation (*t*=12 h). Arrow a: peripheral *S. aureus* band, arrow b: middle band of *C. albicans,* arrow c, internal discontinuous band of *S. aureus*. All images are (or are derived from) maximum-intensity z-projections. Scale bar in A, 100 µm; scale bar in B, 1000 µm; scale bar in C, 100 µm. (c) Digital zoom of halo illustrating serrated interface between *C. albicans* and *S. aureus* bands within the halo. White arrows highlight examples of *C. albicans* hyphae penetrating the *S. aureus* band in (**i**) yeast-form *C. albicans/S. aureus* biofilm and (ii) hyphal-form biofilm 9 h post-inoculation (*t*=9 h). Scale bar in (c), 100 µm.

Mesolens images captured over the 12 h time period reveal the emergence of similar segregation of the species within the halo of both biofilms, and distinct morphotype-dependent core structures. Close inspection of cell morphology at *t*=0 indicates the presence of only yeast cell morphotype *C. albicans* and *S. aureus* in both hyphal- and yeast-form biofilm halos [Fig. 2a], whereas the presence of hyphae is only observed in the core of the hyphal-form biofilm [Fig. 1a(i) vs b(i)].

In the early stages of biofilm development, *C. albicans* and *S. aureus* within the halos of both *C. albicans* morphotype biofilms begin to emerge into discrete species-specific banding patterns. These develop into a middle band of *C. albicans* flanked by an outer band of *S. aureus* on the periphery of the biofilm, and a broken discontinuous internal band of *S. aureus* on the core-proximal edge of the halo by 12 h [Fig. 2b]. This structure is first noticeable as an internal band of *C. albicans* cells at the 3 h time point, followed by the emergence of the faster growing *S. aureus* as a band at the periphery by 6 h, as shown in Fig. 1a (ii, iii) and b (ii, iii), respectively. In later stages of biofilm formation at 9 and 12 h post-inoculation, an additional broken band of *S. aureus* emerges at the core-facing edge of the halo [Fig. 1a (iv, v) and b (iv,v)]. At 9 h, a serrated boundary is observed between the internal *C. albicans* band and the peripheral *S. aureus* band, with multiple fingers of *C. albicans* invading the peripheral subpopulation of *S. aureus* [Fig. 2c]. These *C. albicans* incursions begin as hyphae from the *C. albicans* halo band, as their starting width at *t*=6 h is between 2–4 µm (Fig. S2). By 12 h, similar halo banding patterns are observed in biofilms of both *C. albicans* cell morphotypes [Fig. 1a (v) and b (v)].

**Fig. 3.**
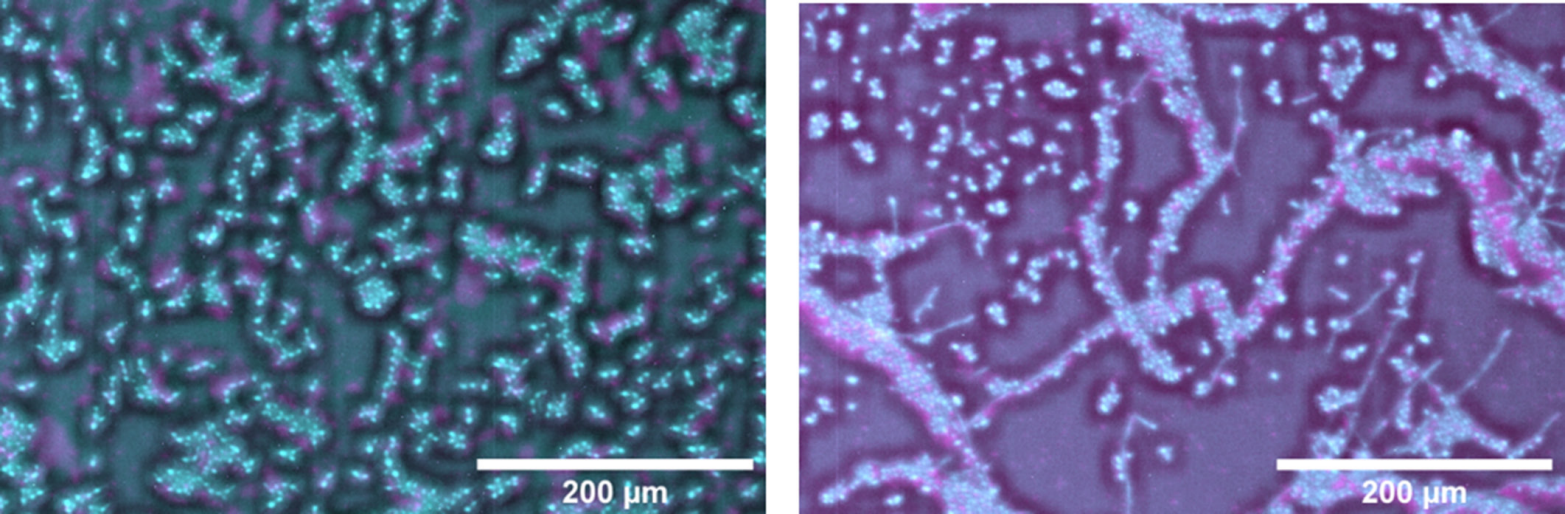
Impact of *C. albicans* cell morphotype on initial cell deposition within biofilm cores of pACT-1 GFP (cyan)*/S. aureus* N315 mCherry (magenta) dual-species biofilms. (**i**) Digital zoom of yeast-form biofilm and (ii) hyphal- form biofilm at *t*=0. Asterisks in (ii) indicate *S. aureus* and yeast-form *C. albicans* accumulation along hyphal–cell aggregates.

Strikingly, the core structure is *C. albicans* cell morphotype dependent. There is a prominent difference in cell deposition between the yeast-form and hyphal-form dual-species biofilm cores, noticeable from the initial time point. In the yeast-form biofilm, cells of both *C. albicans* and *S. aureus* are randomly deposited within the core at deposition [Fig. 3 (i)] However, cells from the hyphal-form culture accumulate in aggregates of *S. aureus* and *C. albicans* yeast-form cells clustered around hyphae, with larger cell-free gaps between these cell groupings. This differential deposition results in the development of two architecturally distinct core structures as the biofilms mature. At 3 h post-inoculation in the yeast-form biofilm, *C. albicans* and *S. aureus* cells begin to develop into discrete microcolonies of each species [Fig. 1a (ii)], which cover the core with a mosaic-like pattern by the 12 h time point [Fig. 1a (v)]. This is in marked difference to the hyphal-form biofilm, where the hyphal–cell aggregates expand into ropes of intercalated *C. albicans* and *S. aureus* over the first 6 h post-inoculation [Fig. 1b (ii)], resulting in a marbled-like distribution of *C. albicans* and *S. aureus* by *t*=12 [Fig. 1b (v)].

### Quantification of cell-derived macrostructures reveals the impact of *C. albicans* cell morphotype on biofilm architecture

**Fig. 4.**
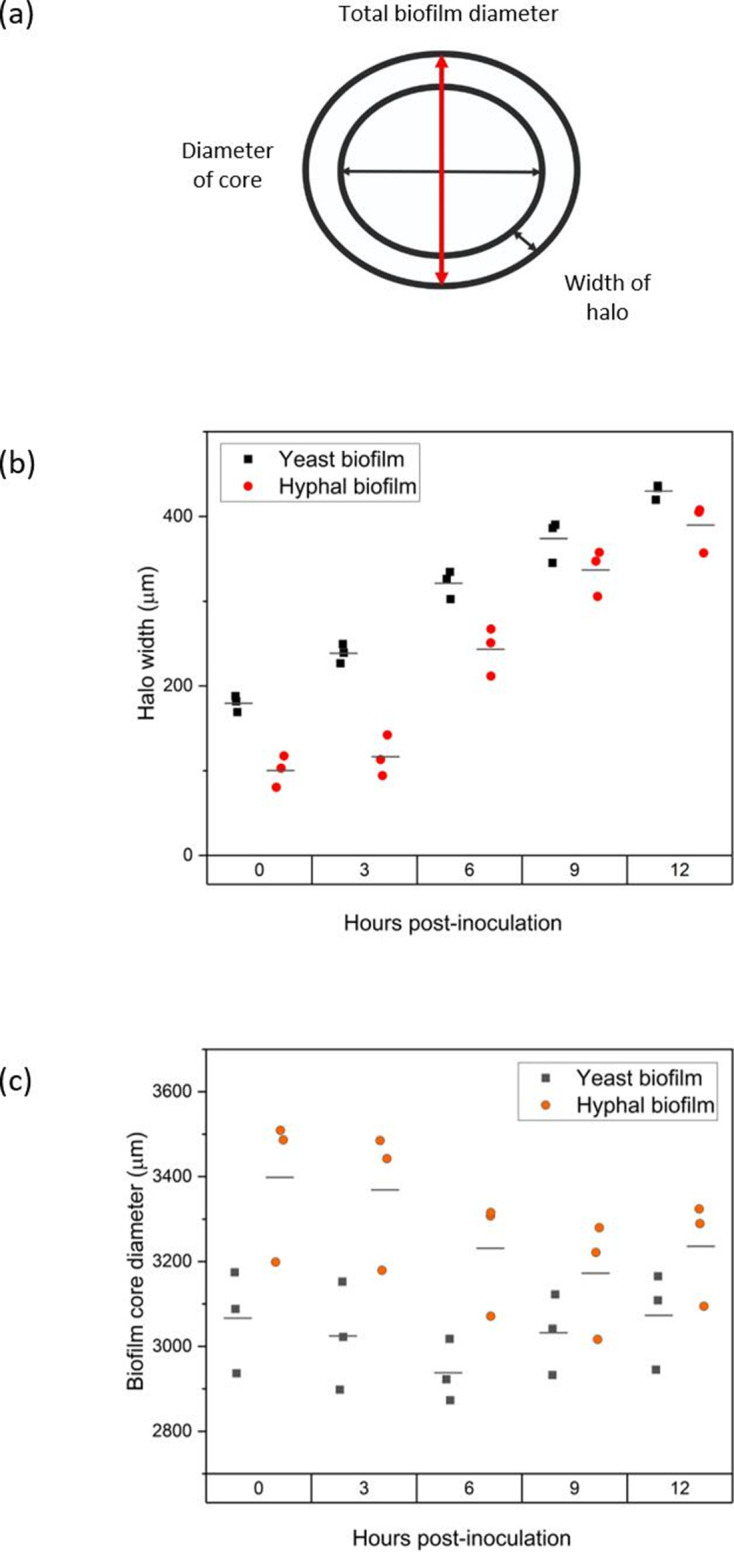
Quantification of yeast-form and hyphal-form biofilm macrostructure properties from time-lapse mesoscopy images. For each time point, three measurements of each property were obtained from three independent biofilms (*n*=3). Standard deviation error bars. (**a**) Diagram of properties measured. (**b**) Halo width of yeast-form (black squares) or hyphal-form (red circles) biofilms. (**c**) Core diameter of yeast-form (black squares) or hyphal-form (red circles) biofilms. *P* values (Supplementary Data) indicate that these results are significant.

Following initial deposition, there are noticeable differences in the cell-derived macrostructures of the hyphal-form biofilm and the yeast-form biofilm in terms of halo width, core diameter and deposition of cells within the core. We quantified these parameters to understand the dynamics of these macrostructures and their development [Fig. 4]. Both types of biofilms have similar total biofilm diameters at all time points [Fig. 1c], averaging 3930 µm (± 106) for yeast-form biofilms and 4033 µm (± 155) for hyphal-form biofilms at the 12 h time point. Biofilm expansion rates are comparable for each biofilm type, averaging between 37–54 µm h^−1^ 3–12 h post-inoculation (Fig. S3, Table S1). The halos of hyphal-form biofilms are noticeably smaller than those of yeast-form biofilms, with an average a width of 100 µm (±13) compared to 180 µm (±7), respectively, at deposition. Differences in halo width between the hyphal-form and yeast-form biofilms decrease over the growth period, although hyphal-form biofilm halos remain narrower than those of yeast-form biofilms, with an average of 390 µm in comparison to the larger yeast-form halo average of 430 µm at 12 h.

As both biofilms have similar total diameters at all time points, we postulated that our findings of a smaller halo width would also imply a larger core diameter, as these two components together compose the total biofilm diameter. Using the same FIJI image analysis method, the quantification of core diameter indicates that this is the case (Fig. 4). The core of hyphal-form biofilms at deposition averages 3398 µm (± 133) versus the smaller yeast-form diameter of 3067 µm (± 87). The Hyphal-form consistently has a larger diameter than that of the yeast-form biofilm across the 12 h. Again, as for the halo data, the difference between core diameters of hyphal-form and yeast-form biofilms does decrease over time, but this difference is still significant (statistical analysis in Table S2).

### Aggregation of free cells by *C. albicans* cells leads to greater interspecies clustering in hyphal-form biofilms

**Fig. 5.**
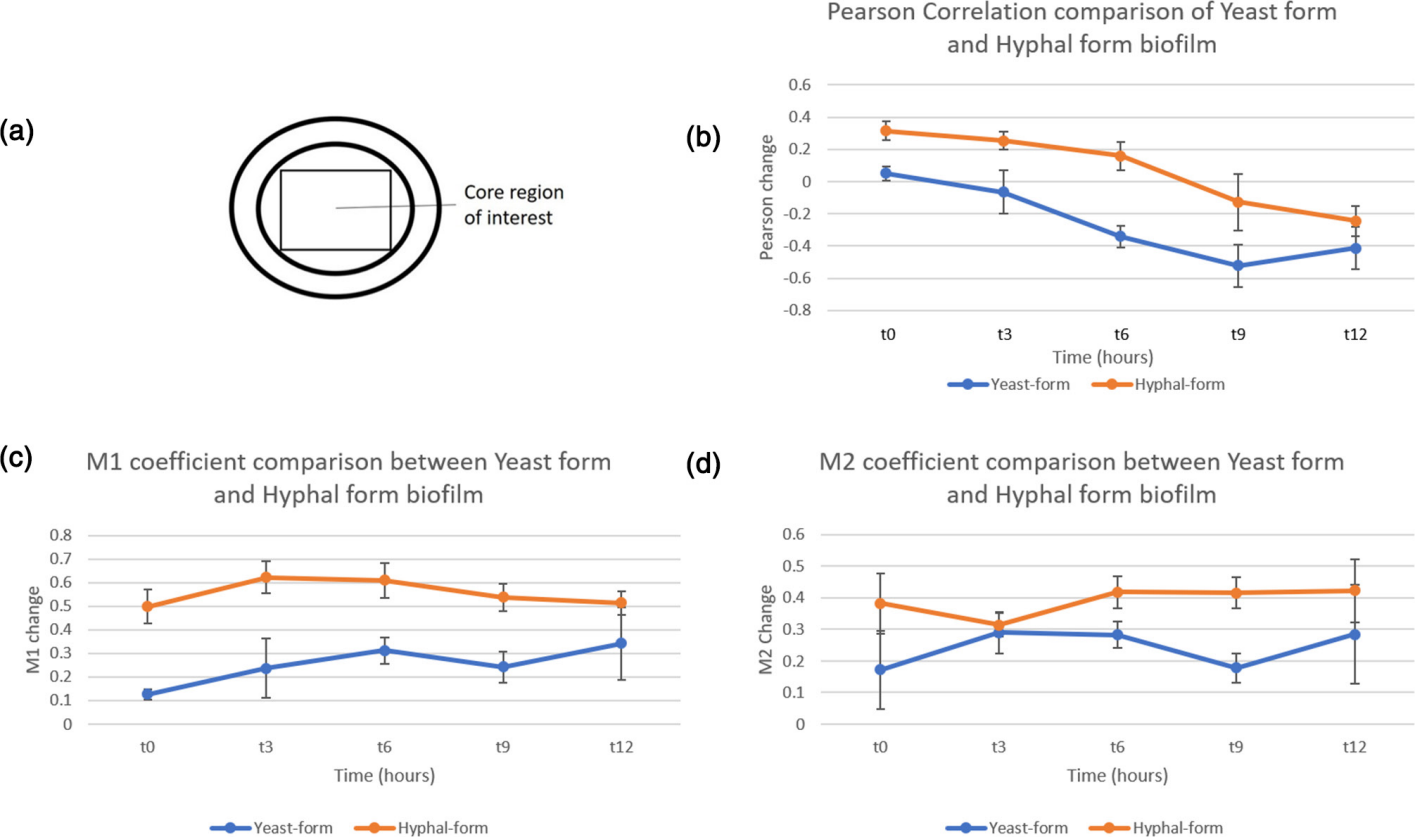
Colocalization of *C. albicans* and *S. aureus* in yeast-form and hyphal-form dual-species biofilms from time-lapse mesoscopy images. (a) Diagram indicating core region of interest selected for colocalization analysis. (b) Comparison of change in PCC between yeast-form (blue) and hyphal-form (orange) dual-species biofilms at each time point, representing the degree of colocalization of *C. albicans* GFP and *S. aureus* mCherry signal. Positive coefficients denote localization and negative coefficients denote inverse localization (i.e. greater signal of one correlating with lower signal of other). (c, d) MOC M1 (overlap of GFP signal with mCherry signal) and M2 (overlap of mCherry signal with GFP signal), respectively, representing the degree of overlap of fluorescent signals. The greater the coefficient value, the more overlap between signals at the same locality. Measurements taken from three independent biofilm cores of each biofilm subtype and error bars are standard deviation at each time point. *P* values (Supplementary Data) indicate that these results are significant.

Due to the correlation of hyphal invasion with systemic *S. aureus* infection [45], we undertook analysis of colocalization of mCherry and GFP signals within biofilm cores to understand the degree of association of *S. aureus* with yeast-form and hyphal-form *C. albicans* in global biofilm structure. In our imaging, we can see an association of *S. aureus* with hyphae at time of deposition, in line with previous findings [27, 29, 46–48]. It was also noted that *S. aureus* accumulate along the edges of hyphal–cell aggregates (Fig. 3i, asterisks). This suggests that there is more to the *S. aureus/C. albicans* physical relationship than *S. aureus*–hyphal interaction alone. It was hypothesized that hyphae may function as a nucleation point for free yeast-form *C. albicans* and *S. aureus* in biofilm as a consequence of fluid dynamics, as particle deposition during droplet evaporation is influenced by particle size, with non-spherical shapes creating localized conditions that cause particles to clump [49]. To quantify this observation of increased association of *C. albicans* and *S. aureus* in the core of the hyphal-form compared to the yeast-form biofilm, PCC and MOC were used to quantify correlation and co-occurrence of both species using the JACop FIJI plugin [40] (Fig. 5a). It was apparent that hyphal-form biofilms have both a higher PCC [Fig. 5(b)] and a higher MOC M1 (Fig. 5c and d) from the deposition point onwards*,* indicating that *C. albicans* and *S. aureus* localize at a greater incidence and for a longer period of time in the biofilm core when hyphal-form *C. albicans* is present. This correlation occurs until 9 h of growth (Table S3). However, this may be influenced by population densities at *t*=12, where both cores are densely packed with both *C. albicans* and *S. aureus* structures*,* and fluorescence may be too great to resolve the relationship. The MOC M2 only shows significant co-occurrence at *t*=9 in comparison to the PCC and MOC M1. As MOC are known to be sensitive to background fluorescence, this is perhaps caused by background levels of our *t*=0 to *t*=6 mCherry images influencing the M2 (mCherry overlap with GFP) to a greater degree than the M1 (GFP overlap with mCherry).

### Hyphal-form biofilms have greater biomass of both *C. albicans* and *S. aureus* than yeast-form biofilms after 9 h of growth

Synergistic interactions between *C. albicans* and *S. aureus* enhance the growth of both organisms, with *S. aureus* growth promoted by *C. albicans* prostaglandin E2 secretion [25], and the induction of *C. albicans* hyphal formation promoted by the presence of *S*. *aureus* cell wall peptidoglycan [50]. It is therefore possible that the increased association of *C. albicans* and *S. aureus* within the hyphal form biofilm may facilitate these synergies and increase growth of either or both organisms. To identify whether growth augmentation occurs in hyphal-form biofilms, the biomass of both yeast-form and hyphal-form biofilms was assessed indirectly by quantifying the percentage change in fluorescence intensity between 3 and 12 h.

**Fig. 6.**
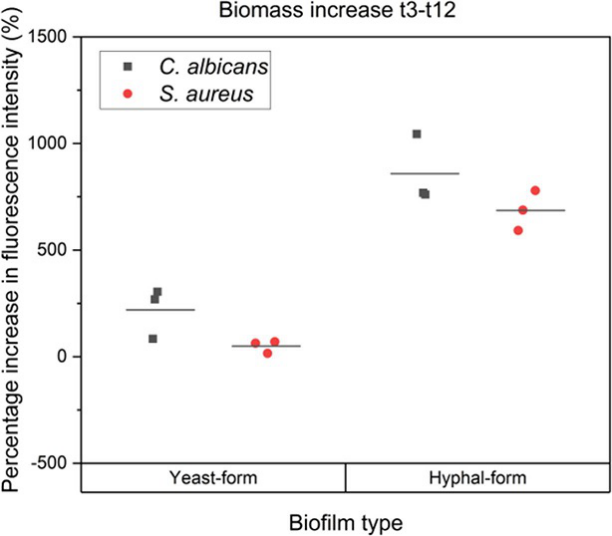
Increase in biomass of yeast-form and hyphal-form biofilms over 9 h . Graph displays indirect measurement of *C. albicans* and *S. aureus* biomass though quantification of percentage increase in fluorescence intensity across t3 to t12 time points. Hyphal-form biofilms show significantly greater biomass increase over the time period compared to yeast-form biofilms.

Data presented in Fig. 6 and Table S4 indicate that greater growth augmentation of both species occurs within the hyphal-form biofilm in comparison to the yeast-form biofilm. The fluorescence intensity of *C. albicans* in the hyphal-form biofilm reveals a fourfold greater increase in biomass in comparison to the yeast-form *C. albicans* population. Strikingly, *S. aureus* shows a starker increase in biomass in the hyphal-form biofilm to that of the *S. aureus* yeast-form biofilm population, with a 10-fold increase in biomass. These results indicate that conditions within a hyphal-form biofilm are significantly more favourable for growth of both *C .albicans* and *S. aureus,* suggesting that interspecies aggregates enhance synergism.

### Biofilm growth confers increased *S. aureus* resistance to vancomycin

It is well documented that association with *C. albicans* increases the resistance of *S. aureus* to antibiotics [18, 26, 29, 51]. To test whether the morphotype of *Candida* present in the biofilms influences antibiotic resistance, the vancomycin sensitivity of both *S. aureus* planktonic culture and *S. aureus* biofilm-derived cells from either yeast and hyphal dual-species biofilms was tested. Biofilms were tested after 6 h of growth, as this is the point where the macrostructures are established.

**Fig. 7.**
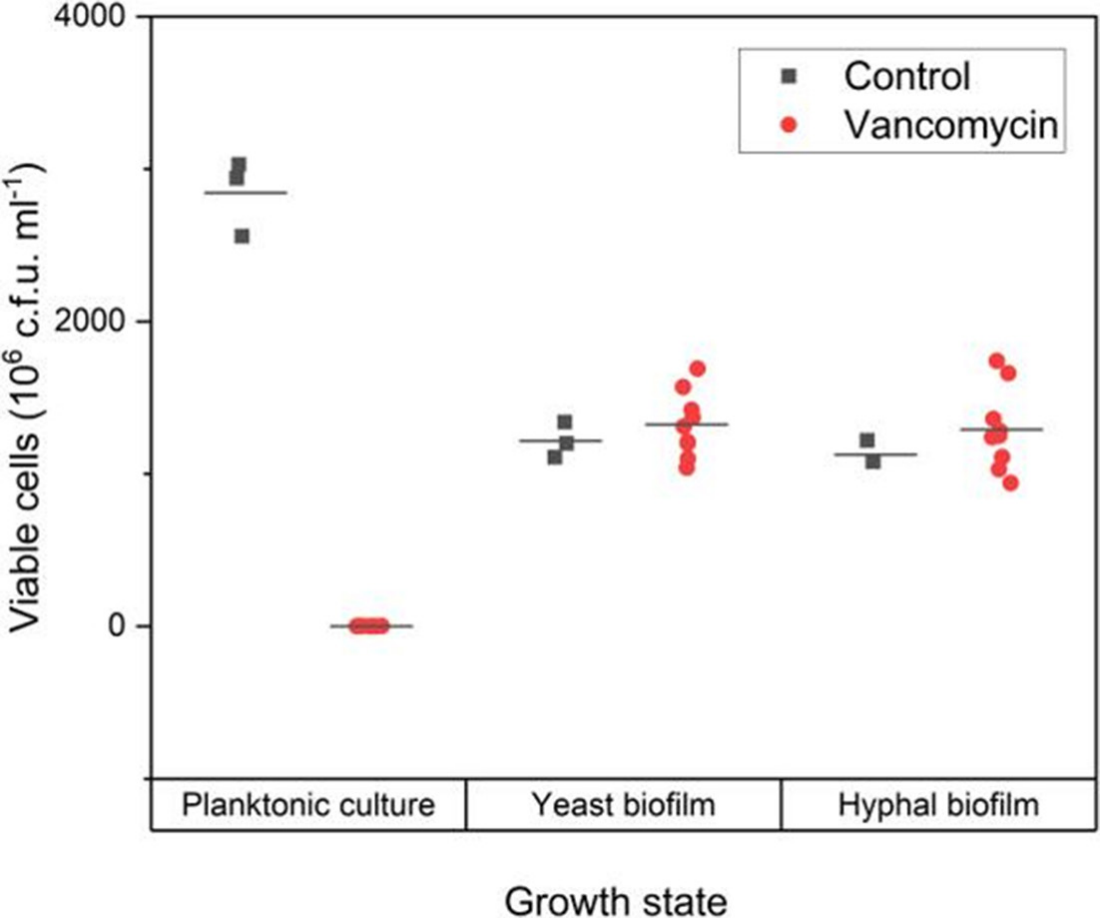
Viability of *S. aureus* after 5 h exposure to vancomycin. Graph displays viable cells determined by c.f.u. count. Planktonic cells used were *S. aureus* biofilm seed cultures. Data presented as 10^6^ c.f.u. ml^−1^. Biofilms of both *C. albicans* morphotypes confer increased resistance to vancomycin in comparison to planktonic culture.

After exposure, the cell viability of planktonic *S. aureus* is reduced significantly by a factor of 3000-fold; in comparison, vancomycin-treated yeast-form and hyphal-form biofilms do not show a reduction in viability, with similar numbers of viable cells with or without vancomycin treatment (Fig. 7, Table S5). These findings confirm that even after only 6 hours, biofilm growth facilitates *S. aureus* resistance to vancomycin when compared to planktonic cultures. No difference was observed between the viability of *S. aureus* in yeast-form and hyphal-form biofilm after vancomycin exposure under these conditions.

## Discussion

Dual-species biofilms are of great importance in the clinic [52, 53]. To determine the role played by the *Candida* morphotype in the development and dynamics of dual-species biofilms a time-lapse mesoscopy method was developed. This enabled the formation of dual-species biofilms produced by *C. albicans* and *S. aureus* to be followed in intricate spatial detail, from deposition of individual cells to mature biofilm structure. This provides an unprecedented level of detail on the emergence of cell-governed macrostrucures within the *C. albicans*/*S. aureus* dual-species biofilm.

These data suggest that the formation of multiple large interspecies clusters by hyphae facilitates interaction of *C. albicans* and *S. aureus* in biofilms from initial deposition onwards. Increased biomass of both *C. albicans* and *S. aureus* in hyphal-form biofilms compared to yeast-form biofilm suggests enhanced metabolic interaction. Although the mechanism underlying growth enhancement in the hyphal-form biofilm was not investigated in this study, there are established interactions of *C. albicans* and *S. aureus* that would influence growth of the reciprocal organism when in close proximity [25, 54]. Similarly, other synergistic events may also be promoted in hyphal biofilms due to increased interspecies clustering. Closer association of *C. albicans* and *S. aureus* in hyphal-form biofilms could result in coating of *S. aureus* cells with *C. albicans* matrix components. This may occur at an earlier stage of biofilm formation in hyphal-form biofilms than in non-hyphal biofilms, affording *S. aureus* the protection of *Candida-*derived β−1,3-glucans against antimicrobial challenge during biofilm formation [26]. Clustering of cells could also expose a larger population of *S. aureus* to metabolites secreted by *C. albicans*, providing opportunity for greater *C. albicans* influence over *S. aureus* through mechanisms such as modulation of the *agr* system through alkalization of the local environment [23, 24]. If indeed other synergistic processes beyond augmentation of growth are promoted in these large interspecies clusters, hyphal-form biofilms may result in a larger population of *S. aureus* with *C. albicans*-augmented virulence and growth, and offer greater opportunity for *S. aureus* hyphal attachment and co-penetration into tissue [27]. However, further study, such as the comparison of other *C. albicans* and *S. aureus* isolates, varying hyphal number of *C. albicans* seed cultures, and altering ratios of *C. albicans* and *S. aureus* founding populations within hyphal-form and yeast-form biofilm, is required to provide further information on the role of interspecies synergy and cell deposition patterns in the heterogeneity of biofilm formation. In addition, these findings suggest that study of yeast- and hyphal-form biofilms by other methods, such as transcriptomic analysis, and further antimicrobial drug challenge over multiple time points during biofilm formation, in the presence of a monospecies *S. aureus* biofilm control, is also required to confirm whether morphotype-specific clusters confer a spatial and/or temporal advantage in dual-species biofilms. Our study tested the minimum concentration of vancomycin required to prevent planktonic growth; however, exposing yeast-form and hyphal-form biofilms to increasing concentrations of vancomycin and at different time points would provide greater insight into the relationship of *C. albicans* cell morphotype, and highlight structurally conferred tolerance to vancomycin

The newly elucidated structural role for *Candida* hyphae in cell deposition patterns and the formation of large interspecies aggregates in biofilm development suggest that, in addition to governing biofilm architecture, these structures may influence inherent antimicrobial resistance of a *C. albicans*/*S. aureus* dual-species biofilm. If these data are reflected in the clinical setting, it is speculated that *C. albicans* cell morphotype plays a greater role in co-infection than simply a point of attachment and port of entry for systemic *S. aureus* infection [46, 55]. If *Candida* hyphae aid the nucleation of planktonic *S. aureus* cells *in vivo* to produce multiple large synergistic foci over a large surface area, as observed here, they may contribute to greater colonization. Over the lifetime of the infection, this could have serious implications for treatment efficacy and patient outcomes.

## Supplementary Data

Supplementary material 1
