# Supplementary material for: Time-lapse mesoscopy of Candida albicans and Staphylococcus aureus dual-species biofilms reveals a structural role for the hyphae of C. albicans in biofilm formation
Source: Microbiology (Reading). 2024 Jan 23;170(1):001426. doi: 10.1099/mic.0.001426 (PMC10866020; doi:10.1099/mic.0.001426)
Supplement: Supplementary material 1 [file mic-170-1426-s001.pdf]

Supplemental data

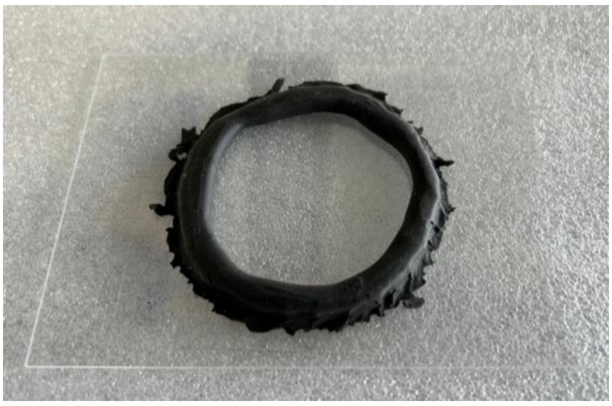

Supplemental Figure 1: Image of Sugru dam manifold for time lapse mesoscopy. Hardened Sugru mouldable glue acts as a dam on a glass coverslip to retain agar without leakage, allowing agar to set into a thin layer for imaging purposes.

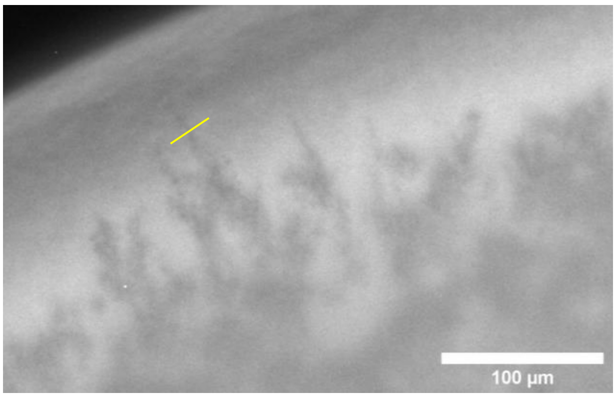

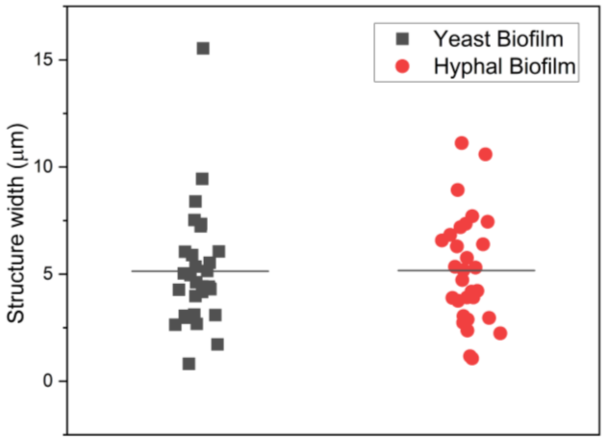

Supplemental Figure 2: Measurement of *C.* albicans projections into *S. aureus* halo. Panel A: Illustration of measurement of *C. albicans* smallest projections using the Fiji line tool. Image is a maximum intensity z-projection from 9 hour time point. Scale bar 100µm. **Graph B:** Widths of *C. albicans* structures in both yeast-form and hyphal-form biofilm halos. Measurements taken from 3 independent experiments.

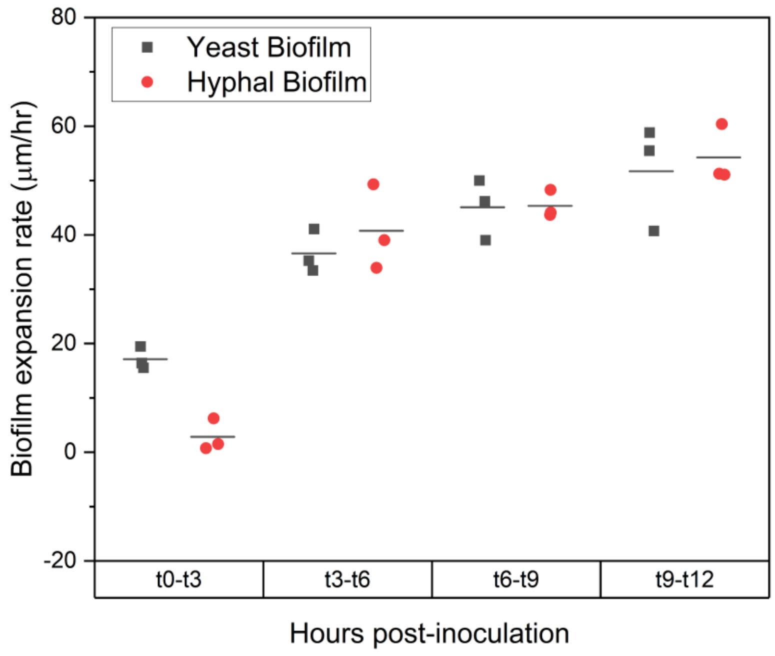

Supplemental Figure 3: Biofilm expansion rates. Graph illustrates biofilm expansion rates of yeast-form biofilm (black squares) and hyphal-form biofilm (red circles) between each 3 hour time point.

Table 1 – Statistical analysis of quantification

Table 2 – Statistical analysis of colocalization

Table 3 – Statistical analysis of percentage increase in fluorescence intensity t3-t12

Table 4 – Statistical analysis of vancomycin treated biofilms

Table 5 – Statistical analysis of biofilm expansion rates

Supplementary table 1: statistical analysis of biofilm expansion rates

**Yeast-form Biofilm**				
	Hours post-inoculation			
	t0-t3	t3-t6	t6-t9	t9-t12
Biofilm 1	19.46467	33.43833	46.19633	40.72867
Biofilm 2	15.53433	35.252	39.03267	55.50133
Biofilm 3	16.404	41.0954	50.01393	58.842
Average	17.13433	36.59524	45.08098	51.69067
Standard deviation	2.064445	4.001363	5.574946	9.639193
P value (T test)	0.012063	0.140551	0.462709	0.341628

**Hyphal form Biofilm**				
	Hours post-inoculation			
	t0-t3	t3-t6	t6-t9	t9-t12
Biofilm 1	1.50467	39.02667	43.65333	51.25033
Biofilm 2	0.75033	33.929	44.081	60.39567
Biofilm 3	6.23833	49.294	48.27267	51.084
Average	2.83111	40.74989	45.33567	54.24333
Standard deviation	2.9747468	7.826105	2.552492	5.328729

Supplementary table 2: statistical analysis of quantification

**Total diameter (µm)**	**Yeast-form Biofilm**					**Hyphal-form Biofilm**				
	Time point					Time point				
	t0	t3	t6	t9	t12	t0	t3	t6	t9	t12
Biofilm 1	3611.717	3670.111	3770.426	3909.015	4031.201	3722.796	3727.31	3844.39	3975.35	4129.101
Biofilm 2	3334.269	3380.872	3486.628	3603.726	3770.23	3382.837	3385.088	3486.875	3619.118	3800.305
Biofilm 3	3488.023	3537.235	3660.521	3810.563	3987.089	3705.838	3724.553	3872.435	4017.253	4170.505
Average	3478.003	3529.406	3639.192	3774.435	3929.507	3603.824	3612.317	3734.567	3870.574	4033.304
Standard Deviation	138.9951	144.7783	143.0962	155.8181	139.6899	191.5678	196.7909	214.9651	218.7726	202.842
P value (T test)	0.062886	0.133474	0.132004	0.11741	0.072096					

**Core diameter (µm)**	**Yeast-form Biofilm**					**Hyphal-form Biofilm**				
	Time point					Time point				
	t0	t3	t6	t9	t12	t0	t3	t6	t9	t12
Biofilm 1	3174.842	3152.717	3017.968	3122.817	3165.587	3486.624	3442.392	3307.667	3221.460	3323.807
Biofilm 2	2936.85	2898.258	2873.452	2932.953	2945.194	3198.962	3179.439	3071.307	3016.623	3094.862
Biofilm 3	3088.439	3022.45	2922.811	3041.885	3109.019	3509.194	3485.348	3315.559	3279.723	3289.545
Average	3066.71	3024.475	2938.077	3032.552	3073.267	3398.26	3369.06	3231.511	3172.602	3236.071
Standard Deviation	120.4747	127.2416	73.45752	95.27548	114.4637	172.9657	165.6149	138.7968	138.1873	123.4849
P value (T test)	0.009695	0.014138	0.017444	0.05201	0.001588					

**Halo width (µm)**	**Yeast-form Biofilm**					**Hyphal-form Biofilm**				
	Time point					Time point				
	t0	t3	t6	t9	t12	t0	t3	t6	t9	t12
Biofilm 1	188.16	249.48	334.607	390.255	433.626	117.367	142.167	250.782	347.263	405.062
Biofilm 2	169.183	226.808	302.544	345.239	419.691	80.434	94.189	211.688	305.612	356.784
Biofilm 3	181.767	239.388	326.449	386.335	436.138	102.989	113.190	267.144	357.576	407.678
Average	179.7033	238.5587	321.2	373.943	429.8183	100.2633	116.5153	243.2047	336.817	389.8413
Standard Deviation	9.655344	11.35873	16.66352	24.93554	8.860005	18.61675	24.16124	28.49393	27.51188	28.65836
P value (T test)	0.002124	0.001925	0.007351	0.00656	0.036659					

Supplementary table 3: Statistical analysis of colocalisation

**Pearsons Coefficient**	**Yeast- form Biofilm**					**Hyphal-form biofilm**				
	t0	t3	t6	t9	t12	t0	t3	t6	t9	t12
Biofilm 1	0.094	0.082	-0.363	-0.519	-0.465	0.28	0.279	0.138	-0.255	-0.258
Biofilm 2	0.01	-0.095	-0.264	-0.391	-0.265	0.279	0.19	0.085	-0.198	-0.331
Biofilm 3	0.047	-0.18	-0.392	-0.656	-0.506	0.381	0.291	0.257	0.072	-0.143
Average	0.050333	-0.06433	-0.33967	-0.522	-0.412	0.313333	0.253333	0.16	-0.127	-0.244
Standard Deviation	0.042099	0.133665	0.067114	0.132525	0.128946	0.058603	0.055175	0.088085	0.17468	0.094779
P value (T test)	0.012755	0.02949	0.014376	0.07137	0.15609					

**Manders Coefficient M1**	**Yeast- form Biofilm**					**Hyphal-form biofilm**				
	t0	t3	t6	t9	t12	t0	t3	t6	t9	t12
Biofilm 1	0.14	0.347	0.311	0.244	0.252	0.521	0.628	0.558	0.47	0.472
Biofilm 2	0.101	0.099	0.369	0.308	0.52	0.419	0.552	0.693	0.563	0.499
Biofilm 3	0.138	0.265	0.254	0.173	0.253	0.555	0.688	0.577	0.579	0.569
Average	0.126333	0.237	0.311333	0.241667	0.341667	0.498333	0.622667	0.609333	0.537333	0.513333
Standard Deviation	0.021962	0.126349	0.057501	0.06753	0.154442	0.070777	0.068157	0.073078	0.058859	0.050063
P value (T test)	0.002997	0.009199	0.003622	0.016909	0.114463					

**Manders Coefficient M2**	**Yeast- form Biofilm**					**Hyphal-form biofilm**				
	t0	t3	t6	t9	t12	t0	t3	t6	t9	t12
Biofilm 1	0.31	0.366	0.316	0.179	0.195	0.282	0.341	0.428	0.363	0.425
Biofilm 2	0.138	0.25	0.296	0.223	0.467	0.391	0.272	0.364	0.422	0.322
Biofilm 3	0.07	0.254	0.238	0.132	0.193	0.474	0.33	0.464	0.461	0.522
Average	0.172667	0.29	0.283333	0.178	0.285	0.382333	0.314333	0.418667	0.415333	0.423
Standard Deviation	0.123699	0.065848	0.040513	0.045508	0.15762	0.096293	0.037072	0.050649	0.049339	0.100015
P value (T test)	0.119737	0.246031	0.051355	0.017814	0.219988					

Supplementary table 4- Statistical analysis of percentage increase in fluorescence intensity t3-t12

**Yeast-form biofilm**			**Hyphal-form biofilm**		
	wavelength (nm)			wavelength (nm)	
	525	635		525	635
Biofilm 1	84.08027	69.89662	Biofilm 1	768.79	779.1121
Biofilm 2	304.9259	63.31025	Biofilm 2	1044.487	687.3211
Biofilm 3	269.3045	15.47839	Biofilm 3	760.6344	591.6985
Average	219.4369	49.56175	Average	857.9704	686.0439
Standard deviation	118.5676	29.7002	Standard deviation	161.5793	93.71334
P value	0.006809	0.001857			

Supplementary table 5: Statistical analysis of vancomycin treated Biofilms

All values are in 10^6 CFU/mL

Untreated controls

	Planktonic *S. aureus*	Yeast-form Biofilm	Hyphal-form Biofilm
	2940	1340	1080
	2560	1200	1220
	3030	1110	1080
average	2843.333	1216.667	1126.667
Standard deviation	249.4661	115.9023	80.82904
P value (t test) against vancomycin treated	0.001278	0.293659	0.013266

Vancomycin treated

	Planktonic *S. aureus*			Yeast-form Biofilm			Hyphal-form Biofilm		
	Isolate 1	Isolate 2	Isolate 3	Biofilm 1	Biofilm 2	Biofilm 3	Biofilm 1	Biofilm 2	Biofilm 3
	1.1	0.646	0.54	1040	1420	1200	1740	1250	1360
	1.24	0.686	1.02	1100	1570	1370	940	1110	1280
	0.52	0.6	0.66	1210	1310	1690	1030	1660	1240
average	0.953333	0.644	0.74	1116.667	1433.333	1420	1236.667	1340	1293.333
standard deviation	0.38175	0.043035	0.2498	86.21678	130.5118	248.7971	438.2161	285.8321	61.10101
P value (t test)				0.003034			0.00027		
